# The influence of gratitude on pre-service teachers’ career goal self-efficacy: Chained intermediary analysis of meaning in life and career calling

**DOI:** 10.3389/fpsyg.2022.843276

**Published:** 2022-07-28

**Authors:** Sensen Zhang, Yulun Tang, Shaohong Yong

**Affiliations:** Institute of Education, Ningxia University, Yinchuan, China

**Keywords:** gratitude, meaning in life, career calling, career goal self-efficacy, pre-service teachers

## Abstract

**Objective:**

The aim of the study was to explore the relationship among gratitude, meaning in life (MIL), career calling, and career goal self-efficacy (CGSE) of the pre-service teachers in the Free Teacher Education program in China and the internal mechanism of action.

**Methods:**

In this study, gratitude, MIL, career calling, and CGSE questionnaires were used to investigate 801 pre-service teachers. IBM SPSS 25.0 and AMOS 24.0 were used for data processing, and SPSS macro program Model 6 was used for the mediating mechanism.

**Results:**

(1) Gratitude was positively correlated with MIL and career calling. MIL was positively correlated with career calling. Gratitude, MIL, and career calling were significantly and positively associated with CGSE. (2) Gratitude influences pre-service teachers’ CGSE mainly through the independent intermediary of MIL and career calling, and the chain intermediary of MIL→career calling, three indirect effects.

**Conclusion:**

Gratitude indirectly predicts CGSE of pre-service teachers not only through the independent intermediary of MIL and career calling but also through the chain intermediary of MIL and career calling.

## Introduction

The quality of rural education threatened by the shortage of teachers is a global problem (Biddle and Azano, 2016); there is a growing awareness that “cross-cutting factors like race, social class, disability, and gender seem inevitably linked to the inequalities in education” (Holsinger and Jacob, 2009). Educational inequality is a long-standing problem that remains widespread around the world, including in the wealthiest countries (Yin, 2021). The school system in urban and rural China is also highly imbalanced (Gao et al., 2016; Li et al., 2020). Teachers are attracted by high-performing schools that are in coastal cities in southeastern China (Lee and Manzon, 2014; Wu, 2020); meanwhile, the urban–rural educational unfairness has been exacerbated by the unequal affordability of higher education (Mok and Jiang, 2018; Hannum et al., 2019).

The Free Teacher Education (FTE) program was piloted by the government of China to attract young graduates to be teachers and to improve education in impoverished rural regions through free college education and guaranteed jobs (China’s Ministry of Education, 2007). It is much like exemplary programs, such as Teach for America, Teach First in Great Britain, and Teach for Australia, aiming to fill vacant teaching positions with qualified teachers to combat educational inequality, which appeal to high-quality university graduates with strong political and social idealism in purpose (Lee and Manzon, 2014; Yin, 2021). With the completion of the initial pilot project, the government expanded it nationwide, increasing financial support and lowering the college entrance examination score requirement for recruitment, while imposing various restrictions on participants. In the extensive implementation process, it seems to be slowly morphing into an economic “carrot and stick” policy, which requires participants to teach in primary or secondary schools guaranteed by the government for 10 years. For urban schools employ those FTE program teachers, they must first serve in rural schools for 2 years. If they break the contract, they must bear the consequences that include refunding educational costs, paying a fine, and being put on a credit blacklist (China’s Ministry of Education, 2011, 2018). Higher education is regarded as a channel to change destiny; pre-service teachers rely heavily on utilitarianism when choosing the FTE program. Due to the duality of guarantee and restriction of the policy, participants could be a teacher as long as successfully recruited. On one hand, participants are not grateful for the policy for providing the opportunity to be pre-service teachers because of the lack of sustainable career development (Lee and Manzon, 2014; Li et al., 2021). On the other hand, the range of career choices is limited, and career goal self-efficacy is directly affected (Kaminsky and Behrend, 2015; Mi, 2020). More seriously, there is no further guidance and cultivation in the school education process to help them explore the meaning of life and the professional mission of being a teacher, harming their expectations as teachers seriously (Wang and Gao, 2013).

In a study of 1800 FTE pre-service teachers, more than 80% wanted to break the contract and be a job-hoper (Zhou et al., 2010); many FTE teachers did not fulfill obligations (Hong et al., 2015; Wu, 2020). Severe public concerns have been raised on the effectiveness of the FTE. Unfortunately, behind these survey statistics, little is known about the psychological mechanism held by FTE pre-service teachers. Career goal self-efficacy (CGSE) is one of the critical components of sustainable career development (Allan and Duffy, 2014). When college students inevitably encounter various difficulties during their study and personal growth periods, such as a low level of gratitude (Wu, 2020), lack of environmental and meaning in life exploration (Su et al., 2014), and lack of necessary occupational skills in the critical stages of determining career development goals (Wang and Gao, 2013), it is required to analyze their inner characteristics and drive their career calling to enhance CGSE (Allan and Duffy, 2014; Lee et al., 2020). An individual’s internal CGSE shows a positive attitude which can help individuals to enhance their confidence in their inner resources and recognition to be a teacher (Zhang et al., 2019b). In summary, this study takes FTE participants to investigate the relationship and mechanism between gratitude and CGSE to help educators understand the gap between policy expectations and reality, and explore practical solutions.

### Gratitude and career goal self-efficacy

Gratitude is a trait to be thankful, involving an appreciation of the social and psychological resources available in a person’s life (Mccullough et al., 2002). It often involves exhibiting prosocial behaviors and attributing credits to others, regardless of personal gain or loss (DeWall et al., 2012). Gratitude is an effective predictor of psychological wellbeing (Davis et al., 2016); in fact, gratitude interventions might be the most effective tools that positive psychology has produced (Wood et al., 2010). Positive social relationships (Wood et al., 2008), desirable social outcomes, social support (Kong et al., 2015), and willingness to strengthen the benefactor’s social ties (Algoe and Haidt, 2009) are closely associated with gratitude. Furthermore, academic achievement (Froh et al., 2011) and autonomous motivation (Valdez et al., 2017; Valdez and Chu, 2020) have also been demonstrated to associate with gratitude. The engine theory of wellbeing divides variables into three categories: dynamic external (e.g., financial resources) and relatively stable internal factors are input variables to promote happiness, and the best psychological functioning and individual decision-making processes and behaviors (e.g., emotional states like gratitude) are shaped by process variables and outcome variables of voluntary behaviors (e.g., to be a teacher) that characterize happiness (Jayawickreme et al., 2012). According to the theory, the FTE program as an external financial resource provides participants, especially families in poor economic conditions, with opportunities to change their life paths through institutional guarantee, if individuals could be appreciated internally and regard becoming a teacher as a career goal, which would increase CGSE. In addition, the starting point of the FTE program is to solve the unbalanced development of regional education, to change the external social motivation into the individual intrinsic motivation, to guide and strengthen the participants’ college life further, which would produce a win–win situation for individuals and society. Similarly, the self-determination model explains how goals are selected along a continuum from external to intrinsic motivation (Deci and Ryan, 1985). Intrinsic or defined goals are self-concordant because they derive from one’s true interests and values (Allan and Duffy, 2014). Over time, people are more likely to pursue self-concordant goals and achieve them with high goal self-efficacy and sustained hard effort (Sheldon and Kasser, 1998). Previous studies have linked self-efficacy beliefs to sustained efforts to goal achievement (Sheldon and Elliot, 1999), goal commitment and progress (Gebhardt et al., 2010). Grouzet et al. (2005) conducted a survey of goal content across 15 cultures with 2,000 participants and grouped items into 11 goals, and the intrinsic (e.g., self-acceptance) versus extrinsic (e.g., financial success) goals were consistently organized in a circumplex fashion, which were not only shared cross-culturally but organized similarly. In general, it is necessary to embrace gratitude because of cultivating people’s positive traits (Mao et al., 2021); when individuals can truly experience gratitude, they tend to think positively of themselves (Toussaint and Friedman, 2009; Mao et al., 2021), boost self-confidence (Jo and Kim, 2019), and positively significantly influence CGSE (Datu and Yuen, 2020; Mi, 2020).

### Gratitude, meaning in life, career calling, and career goal self-efficacy

Meaning in life (MIL) is defined as the existence and nature of reality, meaning, a person’s sense of purpose, and an accompanying sense of achievement (Steger et al., 2006; Steger, 2010). First, gratitude enhances the tendency to live a meaningful life and associate it with life’s goals (Wood et al., 2008). Since gratitude involves the evaluation of positive things one has, and MIL includes the appraisal of the importance of one’s existence, maintaining an appreciative life orientation can affirm one’s existence further (Ryff and Singer, 1998). In addition, recent studies have also shown gratitude can strongly predict MIL (Datu and Mateo, 2015; Fuochi and Voci, 2020; Chih-Che, 2021). Second, MIL can predict CGSE positively. The conservation of resources (COR) theory can explain the relationship (Salanova et al., 2010). A key driver of motivating and sustaining individual behavior is accumulating relationships and personal resources. MIL is helping individuals generate positive attitudes (e.g., a strong sense of self-efficacy) and loyalty behaviors (e.g., be a teacher by contract) to the organization. Furthermore, the COR creates a revenue spiral from initial resource gains to future gains, which means individuals will not only have to protect and maintain existing resources (e.g., they have been a pre-service teacher and been trained for 4 years on purpose) but also strive for more resources (e.g., they are straightforward to become a respected teacher in a populous country), and having MIL means they can get other resources quickly and effectively that lead to more positive results. Disabato et al. (2017) tested a longitudinal mediation model using a sample of 797 participants from 43 different countries where gratitude predicts goal pursuit and MIL mediates the effect. Recent research also showed that MIL could reduce stress and job burnout (Park and Baumeister, 2017; Dulaney et al., 2018) and positively predict life satisfaction, goal self-efficacy, and work performance (Ang, 2012; Lewis et al., 2018; Schippers and Ziegler, 2019; Tilburg and Igou, 2019).

Career calling is a meaningful and prosocial career prompted by an external force as defined by Dik et al. (2009) in brief. On the one hand, there are much direct and indirect empirical evidence that gratitude predicts calling effectively; individuals with high levels of appreciation have a more vital ability to empathize (Mccullough et al., 2002), which makes them perceive social support more likely from others (Wood et al., 2008). In turn, a three-wave longitudinal study documented that one’s calling can be prompted by the perceived social support (Dalla Rosa et al., 2019). In addition, studies have shown that gratitude has a positive impact on people’s wellbeing (Tian et al., 2016) and prosocial motivation (Ma et al., 2017). Calling could be regarded as a form of wellbeing (Leffel et al., 2018), and the relatedness, autonomy, and competence need satisfaction could confirm it (Zhang and Jin, 2019); prosocial motivation has been coincided as its component (Duffy and Dik, 2013). On the other hand, calling is associated with self-efficacy striving, which is a belief in one’s ability to achieve career goals (i.e., CGSE) and career decision self-efficacy (Dik et al., 2008). Furthermore, it is an effective work attitude, related to the meaning and identity of work; some studies have already proved that (Kaminsky and Behrend, 2015). The social cognitive occupational theory (SCCT), based on the social cognitive theory (Bandura, 1986, 2001), could be used to explain the role of self-efficacy in understanding and predicting the development of academic and career-oriented interests (Lent et al., 1994), and suggests that self-efficacy beliefs and outcome expectations are significant in understanding why people choose specific career paths over others, which has been used to predict and understand career interests and goals positively (Fouad et al., 1996; Soresi, 2003; Kaminsky and Behrend, 2015), as well as more distal outcomes like organizational commitment and job satisfaction (Singh et al., 2013; Allan and Duffy, 2014).

Several studies have related a sense of MIL to perceptions of greater calling (Duffy et al., 2012); treating work as a calling can meet several people’s needs with MIL experience (Baumeister, 1991). It is equal to say that calling has captured the essence of putting a “work-as-meaning” standpoint forward that meaningful work could provide a source of MIL (Duffy and Sedlacek, 2007); Zhang et al. (2017) used a time-lagged analysis of 473 Chinese college students to find that MIL significantly predicted increased calling, but the reverse effects of one’s calling as a predictor of self-clarity about one’s future work life or MIL were not confirmed. Recent studies have also reported MIL as a mediator of the relationships between gratitude and career calling in Chinese undergraduates (Li et al., 2021). Carrier calling prevailingly focuses on the positive effects on career development (Zhang et al., 2017). However, explorations of the predictors and emergence of callings are sparse. Similarly, despite gratitude’s importance in positive psychology and the prospect of gratitude in the workplace, few researchers have explored gratitude in organizational or career development contexts (Dik et al., 2015), and even fewer associated it with CGSE; therefore, further broadening research is needed.

### Hypotheses

The relationship among gratitude, MIL, calling, and CGSE is discussed in the previous sections. We aimed to determine the mediating effect of MIL or calling, as well as the chain mediation consisting of MIL→calling, which affects the relationship between gratitude and CGSE. Therefore, we propose four research hypotheses about the psychological mechanism of Chinese pre-service teachers based on recent studies, the engine theory of wellbeing, self-determination model, and social cognitive career theory.

*H1*: Gratitude can predict the CGSE positively.

*H2*: MIL can play a mediating role in predicting gratitude of CGSE.

*H3*: Calling can play a mediating role in predicting gratitude of CGSE.

*H4*: Gratitude can predict CGSE through the chain mediating role of MIL and calling.

To discuss these issues has its theoretical and practical significance. Initially, to explore the psychological process mechanism of gratitude and CGSE of pre-service teachers in China might enrich the relevant theoretical basis; moreover, how to retain pre-service teachers from the FTE program is a key issue to ensure the stability of the program, and it is of greater practical significance to discuss that.

## Materials and methods

### Participants and procedure

This study chose FTE program pre-service teachers as convenience samples in Western China, from 1 Nov to 15 Nov, using classroom collection, and all pre-service teachers older than 18 years were selected as participants. A similar survey had never been used before. All participants were told no compensation, voluntary participation, and the study was only for academic purposes. To ensure the effectiveness, we primarily contacted counselors of each university, who then distributed the scale at a class meeting. The pre-service teachers read a brief invitation, completed the questionnaire, and submitted it separately. Overall, 878 questionnaires were sent out, 801 valid questionnaires were obtained, and an effective recovery rate was 91.23%; among the participants, 612 were female (76.4%) and 189 were male (23.6%). Details are given in Table 1.

**Table 1:** Demographic characteristics of participants.

Demographic variables		*N* (%)	Demographic variables		*N* (%)
Gender	Male	189 (23.6)	Subject classification	Science and Engineering	164 (20.7)
	Female	612 (76.4)		Humanities and Social Sciences	604 (76.1)
Age	18–19	141 (17.6)	Grade	Arts and Sports	26 (3.3)
	20–22	584 (72.9)		One	258 (32.2)
	23–29	76 (9.5)		Two	227 (28.3)
Birthplace	Urban	296 (37.2)		Three	163 (20.3)
	Rural	499 (62.8)		Four	153 (19.1)
Only one child		166 (20.8)			

### Data analysis

To ensure objectivity and authenticity, the questionnaire data were recorded in an electronic version by an author and another checked. In the data processing, first, we used IBM SPSS 25.0 for preliminary data analysis, descriptive statistics, and the reliability and correlation analyses among the variables. Second, confirmatory factor analysis (CFA) was performed for each scale by AMOS 24.0. Finally, the mediating role of MIL and calling in the relationship between gratitude and CGSE was analyzed by SPSS macro program Model 6.^1^

### Assessment of common method variance

We used the Harman single-factor test to examine systematic measurement errors and common methodological biases (Harman, 1976). Exploratory factor analysis (EFA) was performed for all items, including gratitude, MIL, calling, and CGSE in SPSS 25.0. The results showed that six factors had eigenvalues greater than 1, and the first factor explained the variance is 13.4% less than the critical criterion of 40%; although the possibility of CMV does not eliminate confounding, it shows that the CMV might not confound the interpretation of the results (Podsakoff et al., 2003).

### Measures

#### Gratitude questionnaire (GQ-6)

The gratitude questionnaire was developed by Mccullough et al. (2002) and translated by Li et al. (2012) into Chinese. The participants were asked to respond to statements (e.g., “I have a lot to be thankful for in my life”) on a 7-point Likert scale (1 = strongly disagree; 7 = strongly agree). A higher average score by adding all items indicates a higher level of gratitude. The original scale Cronbach’s alpha coefficient is 0.82 (Mccullough et al., 2002); the Chinese version is 0.83 (Li et al., 2012). In our study, it was 0.81. We also tested McDonald’s omega (Hayes and Coutts, 2020) the estimate of ω was 0.82, which showed good reliability and validity. The fitting index of the model was proved to be ideal (RMSEA = 0.072, CFI = 0.969, NFI = 0.965, IFI = 0.969) by CFA in our study.

#### Meaning in life questionnaire (MLQ-10)

This questionnaire developed by Steger et al. (2006), including 10 items and two dimensions that is a sense of seeking meaning and existential meaning, is a 7-point Likert scale (1 = absolutely untrue; 7 = absolutely true). An example of items includes “I know the meaning of my life”; the higher the score is, the higher the MIL. The scale has been widely used in China; the original alpha is 0.87 (Steger et al., 2006), and the Chinese version is from 0.70 to 0.90 (Zhang et al., 2019a; Lin and Shek, 2021; Ding et al., 2022), showing good reliability and validity, and it was 0.79 in our study. The estimate of ω was 0.77, and the fitting index of the model was proved to be ideal (RMSEA = 0.068, CFI = 0.958, NFI = 0.947, IFI = 0.958) by CFA in our study.

#### Career calling scale (CCS-9)

The calling of normal university students’ scale was adopted from Dobrow and Tosti-Kharas (2011) and contains nine items. For some statements (e.g., “I think teaching can make me happy”), the participants were needed to respond on a 5-point Likert scale (1 = strongly disagree; 5 = strongly agree). A higher average score by adding all items indicates a higher level of calling, and the alpha is 0.90. In addition, this scale has been translated into Chinese and used frequently; Cronbach’s alpha was from 0.85 to 0.95 (Guo et al., 2014; Peng et al., 2020; Zhu et al., 2021). In our study, it was 0.88. The estimate of ω was 0.90, and the fitting index of the model was proved to be ideal (RMSEA = 0.008, CFI = 0.956, NFI = 0.950, IFI = 0.956) by CFA in our study.

#### Career goal self-efficacy scale (CGSS-9)

The scale was developed by Allan and Duffy (2014), based on Dik et al. (2008) career development strivings. It asks participants to respond to five long-term or short-term career goals they are trying to achieve, and items are rated on a 5-point Likert scale how confident they are (1 = not at all confident; 5 = completely confident). Examples of items include “improve my teaching skills” and “enhance my professional knowledge.” The average score is calculated by adding all items to give the CGSE score, and Cronbach’s alpha is 0.74. Li (2020) tested the reliability and validity in China, and the alpha was 0.929, respectively. In our study, it was 0.88. The estimate of ω was also 0.88, and the fitting index of the model was proved to be ideal (RMSEA = 0.035, CFI = 0.978, NFI = 0.976, IFI = 0.978) by CFA in our study.

## Results

### Descriptive statistics and correlation

As expected, gratitude, MIL, and calling were correlated with CGSE significantly. However, MIL and calling also had a significant positive correlation. Moreover, MIL and calling positively correlated significantly with gratitude (see Table 2 for more details).

**Table 2:** Means, standard deviations, and correlations among major variables.

Variable	M	SD	1	2	3	4
1. Gratitude	5.91	0.81	1			
2. Meaning in life	5.18	0.76	0.300^**^	1		
3. Career calling	3.85	0.63	0.195^**^	0.388^**^	1	
4. Career goal self-efficacy	4.08	0.57	0.314^**^	0.440^**^	0.525^**^	1

*N = 801*.

** *p < 0.01*; all tests were two-tailed.

### Chain mediation model analysis

As shown in Table 2, correlation analysis between variables meets the requirements for the further mediating effect of statistical analysis (Hayes, 2012). After controlling for demographic characteristics of the participants, the mediating role of MIL and calling in the relationship between gratitude and CGSE was analyzed by SPSS macro program Model 6 (see Footnote 1).

**Figure 1:**
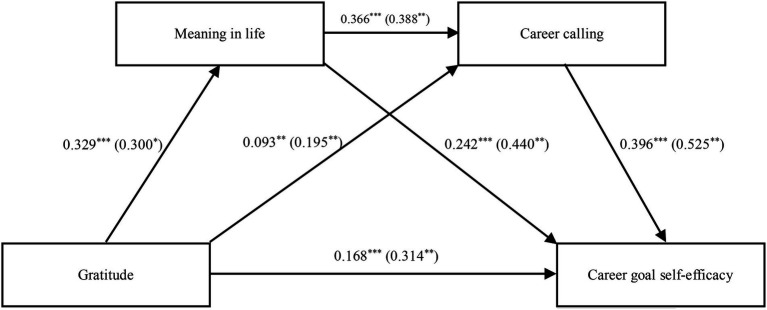
Chain mediation model. It shows the effects of gratitude, meaning in life, and calling on career goal self-efficacy. Within parentheses, we present path coefficients for the direct effects before the mediator was entered. The regression coefficient was obtained after controlling demographic variables in the SPSS macro program Model 6. *N* = 801; ^***^p < 0.001, ^**^p < 0.01, and ^*^p < 0.05.

With demographic variables as control variables, Table 3 shows the regression analysis results, which showed that gratitude positively predicts CGSE (*β* = 0.332, *p* < 0.001). H1 was proved. When MIL and calling were included, gratitude significantly predicts MIL (*β* = 0.329, *p* < 0.001) and calling (*β* = 0.093, *p* < 0.01). MIL significantly predicted calling (*β* = 0.366, *p* < 0.001) and CGSE (*β* = 0.242, *p* < 0.001). Moreover, calling predicted CGSE (*β* = 0.396, *p* < 0.001); at this point, the direct effect value of gratitude reduced significantly on CGSE (*β* = 0.168, *p* < 0.001). It followed from these outcomes that MIL and calling mediate significantly among the influences of gratitude on CGSE. H2–4 were tested. Table 4 and Figure 1 show the mediating effect value of MIL and calling between gratitude and CGSE. A bootstrap estimation approach with 5,000 samples was used to test the indirect effects, and the total standardized mediation effect of MIL and calling between gratitude and CGSE was 0.12.

**Table 3:** Regression analysis.

Regression equation		Fitting index			Regression coefficient significance	
Result variable	Predictor variable	R	R^2^	F	**β**	t
Career goal self-efficacy		0.332	0.110	15.808^***^		
	Gender				−0.098	−2.570^**^
	Age				0.012	0.351
	Only one child				−0.041	−1.028
	Birthplace				0.032	0.862
	Subject classification				0.010	0.281
	Gratitude				0.332	9.517^***^
Meaning in life		0.332	0.111	15.883^***^		
	Gender				−0.080	−2.09^**^
	Age				0.049	1.417
	Only one child				−0.011	−0.286
	Birthplace				0.034	0.896
	Subject classification				−0.077	−2.154^**^
	Gratitude				0.329	9.430^***^
Career calling		0.408	0.167	21.869^***^		
	Gender				0.015	0.399
	Age				0.031	0.907
	Only one child				−0.063	−1.656
	Birthplace				0.000	−0.001
	Subject classification				0.003	0.100
	Meaning in life				0.366	10.462^***^
	Gratitude				0.093	2.618^**^
Career goal self-efficacy		0.611	0.374	57.092^***^		
	Gender				−0.073	−2.278^**^
	Age				−0.019	−0.648
	Only one child				−0.011	−0.335
	Birthplace				0.019	0.613
	Subject classification				0.039	1.276
	career calling				0.396	12.642^***^
	Meaning in life				0.242	7.453^***^
	Gratitude				0.168	5.398^***^

N = 801. All variables have been standardized. This table presents the results of the multiple hierarchical regression analyses, demographic variables are the control variables, gratitude is an independent variable, and CGSE is the result variable.

** *p *< 0.01;

*** *p *< 0.001.

**Table 4:** Mediation effect analysis.

	Indirect effect	Boot SE	Boot LLCI	Boot ULCI	Ratio of indirect to total effect
Total indirect effect	0.115	0.017	0.084	0.148	49.45%
Indirect effect 1	0.056	0.011	0.036	0.078	23.94%
Indirect effect 2	0.026	0.011	0.005	0.05	11.15%
Indirect effect 3	0.033	0.006	0.022	0.047	14.36%
Compare 1	0.03	0.017	−0.004	0.063	
Compare 2	0.022	0.012	−0.002	0.047	
Compare 3	−0.007	0.013	−0.032	0.018	

Boot SE and Boot LLCI and ULCI limits, respectively, refer to the standard error of indirect effects and lower and upper limits of 95% confidence interval estimated by the offset-corrected percentile bootstrap method. Indirect effect 1: gratitude→MIL→CGSE; indirect effect 2: gratitude→calling→CGSE; indirect effect 3: gratitude→MIL→calling→CGSE.

Specifically, the mediating effect included indirect effects, which were produced by the following three pathways: gratitude→MIL→CGSE, and indirect effect 1 (0.06); gratitude→calling→CGSE, and indirect effect 2 (0.03), and gratitude→MIL→calling →CGSE, and indirect effect 3 (0.03). Table 4 shows that indirect effects 1, 2, and 3 accounted, respectively, for 23.93, 11.15, and 14.36% of the total effects. All indirect effects were significant because the bootstrap 95% confidence interval for all did not contain zero. H2-4 was proved again. The bootstrap 95% confidence interval of comparison 1 showing the difference between indirect effects 1 and 2 contained zero, which indicated no significant difference between them. Similarly, no significant difference was found among paths (See Table 3 and Figure 1 for more details).

These results suggested that gratitude indirectly predicted CGSE not only through the independent mediating effect of MIL and calling but also through the chain mediating effect of MIL and calling. The MIL independent mediating effect accounted for the highest proportion of the total effect (23.94%).

## Discussion

This study constructed a model to explore the mediating role of MIL and calling in the relationship between gratitude and CGSE of the pre-service teachers in China. Our results would help identify the underlying mechanisms of how gratitude influences CGSE, and the conditions under which this effect occurs. These findings have theoretical and practical implications for interventions to improve pre-service teachers’ CGSE.

### Theoretical implications

First, consistent with a prior study (Datu and Yuen, 2020), we found that gratitude is a significant positive predictor of the CGSE of Chinese pre-service teachers, that is, gratitude is an important incentive for pre-service teachers’ CGSE in the Chinese context. When the FTE participants believe that the program is a kind of social support and positive social relationship and are willing to tie with the benefactor closely, a high level of gratitude was associated with increased self-efficacy in career and academic development; it is reasonable to assume that understanding the FAE program properly and connecting with educators closely may shape the gratitude and CGSE beliefs of pre-service teachers; therefore, it is crucial for educators to purposefully guide and cultivate that during their college period.

It is interesting to investigate the internal process mechanism of how CGSE might benefit from gratitude, to attain positive results. This study explored the relationship between gratitude and CGSE from different perspectives. We found that MIL is a mediator of gratitude, affecting CGSE for the first time, which means gratitude can promote CGSE by enhancing MIL. There is empirical evidence that gratitude has a positive effect on CGSE in both Chinese and Western adults, which is consistent with the theoretical views of some scholars (Datu and Yuen, 2020; Mi, 2020).

In addition, the theory of broaden-and-build holds that gratitude builds resources by broadening their scope of thought and action, thus promoting individual growth and development (Fredrickson, 2013). Consistent with this hypothesis, our study found that the effect of gratitude on CGSE was moderated by the meaning of life, which could be thought of as a personal resource that individuals build up from broadened mindsets. This result is consistent with that of previous empirical studies, revealing a positive association between gratitude and MIL (Datu and Mateo, 2015; Mao et al., 2021) and the positive effect of MIL on CGSE (Lewis et al., 2018; Chih-Che, 2021). Confucianism is the dominant philosophy that affects teaching and learning in China; its principles not only emphasize equity and quality of education but also suggest that people should learn to be grateful, love the family, love the country, and perform prosocial behavior in daily life to explore the meaning of life and realize their value rather, than educate itself (Mu et al., 2013). Therefore, it is not only necessary to use a “carrot and stick” policy to attract and constrain the FTE participants’ behavior but also to pay attention to their positive traits like cultivating, exploration abilities, and CGSE in advance.

In the mechanism of gratitude affecting the CGSE, our findings showed that calling is a vital mediating variable from a positive perspective that verifies gratitude can enhance CGSE by promoting calling. First, we found that gratitude promotes calling. As previous studies have shown, gratitude is an important incentive resource that could enhance calling (Mccullough et al., 2002; Wood et al., 2008; Dalla Rosa et al., 2019); our study confirms this relationship. A high level of gratitude has a positive influence on pre-service teachers’ wellbeing and strengths in prosocial motivation, which coincided with the calling component. At the same time, our findings showed that calling is positively correlated with CGSE. When someone with calling is following goal self-efficacy, it may lead to confidence in achieving career goals. People confident in their ability to achieve their career goals are likely to be effective, which is also associated with increased life satisfaction (Allan and Duffy, 2014). Furthermore, pre-service teachers’ calling during their study and personal growth period might change, even dramatically, and then stabilize in adulthood or during the first experiences of work socialization (Vianello et al., 2019). As pre-service teachers are in a highly malleable and significant life stage of calling (Praskova et al., 2014), it is crucial to explore and cultivate predictors closely related to the calling experience. In particular, pre-service teachers are more likely to have a strong calling and may be more likely to have a clear work goal or direction.

Finally, we found that the chain mediation from MIL to calling is also an important path that gratitude affects CGSE. This result indicates that gratitude can improve the MIL, and then MIL enhances pre-service teachers’ calling to promote the CGSE further. The process of psychological mechanisms is relatively complex. Although this study has found the independent mediating effects and the mediating chain effect of MIL and calling, all of which produce partial mediating effects, they cannot fully explain the relationship and other factors which might be worth further exploration during the study period. Furthermore, we found that gratitude→ MIL→ CGSE had the highest indirect effect, accounting for 23.94% of the total effect, an important “motivator” path to promote pre-service teachers’ CGSE. Some scholars believe that emphasizing personal meaning and purpose is a feature of individualism (Steele and Lynch, 2013). This result suggests that in the collectivist FTE policy system, MIL still positively predicts CGSE simultaneously. More importantly, it reflects the integration of individualism and collectivism in the Chinese culture, particularly in the career development of pre-service teachers.

### Practical implications

This study can provide implications for the effective promotion of CGSE. First, gratitude can predict CGSE of pre-service teachers directly. Therefore, the FTE program in China can learn from the recruitment strategy of Teach for America (Hong et al., 2015); when recruiting FTE program participants, it can similarly emphasize its purpose of social change and educational equality and implement more rigorous screening procedures to assess the prosocial attitudes and social commitment. Furthermore, according to our study, pre-service teachers could be screened again in schools, and related policies should be formulated to accommodate participants who have a low level of gratitude and CGSE indeed, which is missing at present. Second, gratitude can affect the CGSE of pre-service teachers through MIL, calling, and the intermediary chain between these two factors, that is, the MIL and calling are the key factors affecting pre-service teachers’ CGSE. First of all, we should pay attention to the psychological resource construction of pre-service teachers; educators need guidance on gratitude, MIL, and pre-service teachers’ career planning and exploration abilities in advance; however, the FTE college curriculum seems to ignore that. In addition, pre-service teachers’ cognition and attitude toward future work should be paid attention; only by helping them have positive beliefs and understanding toward future work value can calling be enhanced to attain CGSE and sustainable career development.

### Limitations and future research directions

First, because of using a cross-sectional study design, it is difficult to determine causality. Therefore, in future studies, the causal relationship between variables could be further studied by experimental or longitudinal design. Second, we only surveyed pre-service teachers in Western China, and the sampling method might introduce a potential bias, although participants were from various schools. Further research should take a random sample from across China or other countries so that the results would be applied worldwide. Finally, using a self-reported questionnaire might have a social approval effect. Future studies can collect data from multiple information sources to ensure the objectivity and authenticity of respondents’ answers.

## Conclusion

The purpose of this study was to explore the relationship between gratitude and CGSE and the underlying mechanism of pre-service teachers in China. We constructed a chain mediation model and found that gratitude can predict not only CGSE directly but also the independent mediating roles of MIL and calling indirectly. Simultaneously, it can also predict CGSE through the chain mediating effect of MIL and calling indirectly.

## Data availability statement

The raw data supporting the conclusions of this article will be made available by the authors, without undue reservation.

## Ethics statement

The studies involving human participants were reviewed and approved by the Ethics Committee of Ningxia University. The patients/participants provided their written informed consent to participate in this study.

## Author contributions

SY proposed, revised, and perfected the hypotheses. SZ analyzed the data and translated and completed the paper. YT collected and revised the data. All authors contributed to the article and approved the submitted version.

## Funding

We would like to thank the National Natural Science Foundation of China (71561021) for the financial support of this study.

## Acknowledgments

We would like to thank editors and reviewers for their critical comments and suggestions. Additionally, we gratefully acknowledge the mathematical statistics knowledge of Ding Fengqin (Ningxia University) and her selflessness and kindness.

## Conflict of interest

The authors declare that the research was conducted in the absence of any commercial or financial relationships that could be construed as a potential conflict of interest.

## Publisher’s note

All claims expressed in this article are solely those of the authors and do not necessarily represent those of their affiliated organizations, or those of the publisher, the editors and the reviewers. Any product that may be evaluated in this article, or claim that may be made by its manufacturer, is not guaranteed or endorsed by the publisher.

## Footnotes

^1^ http://www.afhayes.com
